# Task-Based Image Quality Assessment Comparing Classical and Iterative Cone Beam CT Images on Halcyon^®^

**DOI:** 10.3390/diagnostics13030448

**Published:** 2023-01-26

**Authors:** Marion Lassot-Buys, Rodolfe Verstraet, Djamel Dabli, Gilles Moliner, Joël Greffier

**Affiliations:** 1 Department of Oncology Radiotherapy, Nîmes University Hospital, University Federation of Radiation Oncology of Mediterranean Occitanie, Bd Prof Robert Debré CEDEX 9, 30029 Nîmes, France; 2 Medical Physics Unit, Nîmes University Hospital, Bd Prof Robert Debré, CEDEX 9, 30029 Nîmes, France; 3 Department of Medical Imaging, Nîmes University Hospital, IMAGINE UR UM 103, Montpellier University, 30029 Nîmes, France; 4 Cancer Institute of Montpellier, INSERM U1194 IRCM, 34090 Montpellier, France

**Keywords:** Cone Beam CT, iterative reconstruction, image quality, task-based image quality assessment, radiotherapy

## Abstract

Background: Despite the development of iterative reconstruction (IR) in diagnostic imaging, CBCT are generally reconstructed with filtered back projection (FBP) in radiotherapy. Varian medical systems, recently released with their latest Halcyon^®^ V2.0 accelerator, a new IR algorithm for CBCT reconstruction. Purpose: To assess the image quality of radiotherapy CBCT images reconstructed with FBP and an IR algorithm. Methods: Three CBCT acquisition modes (head, thorax and pelvis large) available on a Halcyon^®^ were assessed. Five acquisitions were performed for all modes on an image quality phantom and reconstructed with FBP and IR. Task-based image quality assessment was performed with noise power spectrum (NPS), task-based transfer function (TTF) and detectability index (d’). To illustrate the image quality obtained with both reconstruction types, CBCT acquisitions were made on 6 patients. Results: The noise magnitude and the spatial frequency of the NPS peak was lower with IR than with FBP for all modes. For all low and high-contrast inserts, the values for TTF at 50% were higher with IR than with FBP. For all inserts and all modes, the contrast values were similar with FBP and IR. For all low and high-contrast simulated lesions, d’ values were higher with IR than with FBP for all modes. These results were also found on the 6 patients where the images were less noisy but smoother with IR-CBCT. Conclusions: Using the IR algorithm for CBCT images in radiotherapy improve image quality and thus could increase the accuracy of online registration and limit positioning errors during processing.

## 1. Introduction

Radiotherapy has been a constantly-evolving field since its establishment at the beginning of the 20th century. Initially, control imaging was only available with a high energy megavoltage (MV) beam [1]. It was only in the 2000s that the first kilovoltage (kV) imagers appeared. Since then, kV imaging has become an integral part of routine radiation therapy. Indeed, cone beam computed tomography (CBCT) kV- is performed weekly or even daily depending on the sites treated, as it allows the online registration of the patient in all three spatial dimensions, according to the images of the day [2].

Conventionally, kV-CBCT is reconstructed by filtered back projection (FBP). However, this method induces a high level of noise and known artefacts [3]. Varian medical systems, with their latest software versions and their Halcyon^®^ V2.0 accelerator, offer “iCBCT”: cone beam computed tomography obtained with iterative reconstruction (IR). Eleven acquisition modes are available, with two possible reconstructions.

IR already exists in diagnostic imaging (computed tomography (CT), nuclear medicine). It is based on a different approach from that of FBP, working with successive approximations leading to the closest solution to the real object. With each iteration, a new slice is estimated. However, the properties of these new algorithms make the spatial resolution dependent on contrast and dose, and change both the noise magnitude and the image texture [4,5,6]. These properties require the use of appropriate metrics to evaluate image quality [4,5,7,8,9].

First, there is the analysis of the Noise Power Spectrum (NPS), whose shape, determined by the spatial frequency of the NPS peak and/or the average spatial frequency of the NPS curve, qualifies the noise texture. Second, the spatial resolution is assessed by the Modulation Transfer Function (MTF), which describes the contrast recovery of the system according to spatial frequency. MTF is only applicable for shifting invariant linear imaging system due to its equation. In the case of the IR algorithm, the imaging system is nonlinear, which means that the contrast of the object and the level of background noise can affect the resolution of the system. This has now become more “task specific” and is called the Task-based Transfer Function (TTF). Third, the detectability index is calculated from TTF and NPS measurements, combined with a description of the imaging task. This metric is a model observer i.e., it is specific to a clinical situation. There are several observer models in the literature [4,10,11] whose properties vary according to their complexity and ability to predict imaging performance in agreement with human observers. The detectability index quantifies the detection performance of the analysis model.

Numerous studies have shown that IR can improve the quality of conventional CT images by reducing the image noise and improving the contrast-to-noise ratio [5,6,7,12,13,14,15,16,17]. Using this type of reconstruction algorithm for CBCT images in radiotherapy could reduce the image noise and improve the technologists’ confidence in online registration of the patient with the CBCT vs reference planning CT.

In this context, the purpose of this study was to assess the image quality of iCBCT, compared to conventional CBCT using these new metrics. By this means, we were able to study the complex image quality properties of IR which might invalidate predictions based on classical metrics [4,5,8,9].

## 2. Materials and Methods

### 2.1. CBCT Acquisitions on the Halcyon^®^ Imaging System

Halcyon^®^ V2.0 is a fast O-ring linear accelerator with a single energy 6 MV flattening filter-free beam and a digital megavoltage imager in the beam path for portal dosimetry and MV imaging. For kV imaging, there is another system, composed of a kV source with a permanent half bow-tie filter. The panel is a gadolinium oxysulfide scintillator (Gd_2_O_2_S) measuring 43 cm × 43 cm which has 1280 × 1280 pixel resolution with a frame rate of 30 images per second. The latter can only provide CBCT acquisition in clinical mode, with a scan range of 245 mm and a scan diameter of 491 mm. 

Eleven CBCT acquisition modes are available depending on the anatomical location (pelvis, breast…) or patient type (adults and pediatrics), with certain parameters defined by default.

### 2.2. Acquisition and Reconstruction Parameters

Acquisitions were performed on the Catphan 504 phantom (The Phantom Laboratory) positioned in the air at the head of the table, for three CBCT acquisition modes (Head, Thorax and Pelvis Large). For each CBCT mode used, five acquisitions were performed with the predefined tube voltage, tube current, exposure time, volume CT dose index (CTDI_vol_) and field of view (FOV) (Table 1).

All images were reconstructed with a default slice thickness of 1.991 mm, a matrix size of 512 × 512 pixels using the FBP and IR algorithms available on the Halcyon^®^ V2.0.

### 2.3. Data Analysis

The reconstructed images were analyzed using iQMetrix-CT software [18], with which a task-based image quality assessment can be made by computing the TTF, the NPS and the detectability index. The methodology used by this software is well-known [18] and has been cited in several publications [19,20,21,22].

#### 2.3.1. Task-Based Transfer Function 

**Figure 1:**
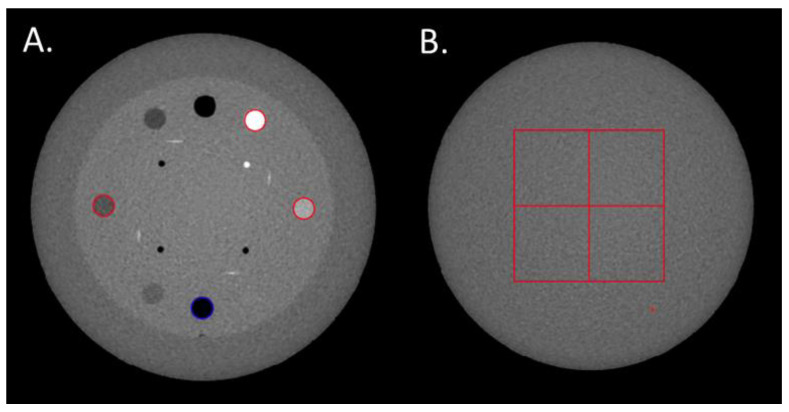
(**A**) Regions of Interests (ROIs) placed on the Delrin, LDPE, Teflon and air inserts to compute the Task-based Transfer Function. (**B**) The four ROIs used to calculate the Noise Power Spectrum.

The TTF was computed in the CTP404 module of the phantom using four cylindrical inserts to assess the spatial resolution according to low (Delrin and LDPE) and high (air and Teflon) contrast conditions. A circular ROI was positioned on each insert (Figure 1A) and the circular-edge technique was used to measure the edge spread function (ESF) [8]. The ESF was obtained by calculating the radius of each pixel from the central pixel of the insert. The line spread function (LSF) was obtained by derivation from the ESF. The TTF was then computed from the LSF normalized Fourier transformation. To minimize the image-noise effect, the TTF was computed from 40 consecutive axial slices (8 slices for each of the 5 acquisitions).

TTF values at 50% (TTF_50%_, mm^−1^) were used to quantify the changes in spatial resolution. The contrast value between each insert and the phantom background computed during the TTF calculation process by the iQMetrix-CT software were also studied.

#### 2.3.2. Noise Power Spectrum

The NPS is equal to the Fast Fourier transform of the image of a uniform object, measured in three dimensions of space and is computed as follows:

(1) $$NPS_{2D}\left(f_{x},f_{y}\right)=\frac{\Delta_{x}\Delta_{y}}{L_{x}L_{y}}\frac{1}{N_{ROI}}\overset{N_{ROI}}{\underset{i=1}{\sum }}{\left|FFT_{2D}\left\{ROI_{i}\left(x,y\right)-FIT_{i}\left(x,y\right)\right\}\right|}^{2}$$

where Δ*_x_* and Δ*_y_* are the pixel size in the x- and y-directions (mm), respectively; *FFT* is the Fast Fourier Transform; *L_x_* and *L_y_* are the lengths of the ROIs in pixels in the x- and y-directions; *N_ROI_* is the number of ROIs; $ROI_{i}\left(x,y\right)$ is the pixel values for the ith *ROI* at the position (x, y) and $FIT_{i}\left(x,y\right)$ is a 2nd order polynomial fit of $ROI_{i}\left(x,y\right)$.

The square root of the area under the NPS curve was used to quantify the noise magnitude. To quantify the changes in noise texture, the spatial frequency of the NPS peak(s) (f_peak_, mm^−1^) was measured.

The NPS was computed on the uniform section of module CTP486 in 85 consecutive axial slices (17 slices for each of the 5 acquisitions) by placing four square ROIs of 46 × 46 pixels for pelvis and thorax modes and 82 × 82 pixels for head mode (Figure 1B).

#### 2.3.3. Detectability Index

A non-prewhitening observer model with eye filter (d′_NPWE_) was used to calculate the detectability index (d′) for four clinical tasks as follows [9]:

(2) $${d^{\prime }}_{NPWE}^{2}=\frac{\left[\iint \left|W\left(u,v\right)\right|².TTF{\left(u,v\right)}^{2}.E{\left(u,v\right)}^{2}dudv\right]²}{\iint {\left|W\left(u,v\right)\right|}^{2}.TTF{\left(u,v\right)}^{2}.NPS{\left(u,v\right)}^{}.E{\left(u,v\right)}^{4}dudv}$$

where *u* and *v* are the spatial frequencies in the x- and y-directions, *E* the eye filter that models the human visual system sensitivity to different spatial frequencies, and *W(u,v)* the task function defined as:

(3) $$W=\left|F\left\{h_{1}\left(x,y\right)-h_{2}\left(x,y\right)\right\}\right|$$

where F the Fourier transform, $h_{1}\left(x,y\right)$ and $h_{2}\left(x,y\right)$ corresponding to the object present and the object absent hypotheses, respectively [12]. The eye filter was modeled according to the visual response function [23].

In accordance with the contrast of lesions found in clinical routine, four task functions of 10-mm diameter with contrasts like the contrast of the four inserts used. These four task functions simulated the clinical low and high contrast lesions treated in radiotherapy.

Interpretation conditions for the d’ calculation were a viewing distance of 500 mm, a zoom factor of 1.5 and a 0.2 mm pixel pitch display.

### 2.4. Patients

To illustrate the image quality obtained with both reconstruction types, CBCT acquisitions were made on 6 patients: 3 for head and neck treatment and 3 for prostate treatment. For the 6 patients, acquisitions were performed two days running during treatment: the first day using FBP and the second day using IR. The same windowing and matching were used to compare CBCT images.

This retrospective study was approved by the local institutional review board (Interface Recherche Bioethique Institutional Review Board, number 22.12.02). The requirement for written informed consent was waived. According to the current regulation, a letter of non-opposition was sent to all patients to inform them of the study and ensure that they did not object to the use of their anonymized data for research studies. No patients informed us of their opposition to their inclusion in the study.

## 3. Results

### 3.1. Noise Power Spectrum

Values of noise magnitude and noise texture for all CBCT modes and both reconstruction types are depicted in Table 2. For all CBCT modes, the noise magnitude was lower with IR than with FBP. The noise magnitude was reduced by −35% for head mode, −19% for thorax mode and −25% for pelvis large mode. For both reconstruction types, the highest noise magnitude values were found for head mode and the lowest values for pelvis large mode.

**Figure 2:**
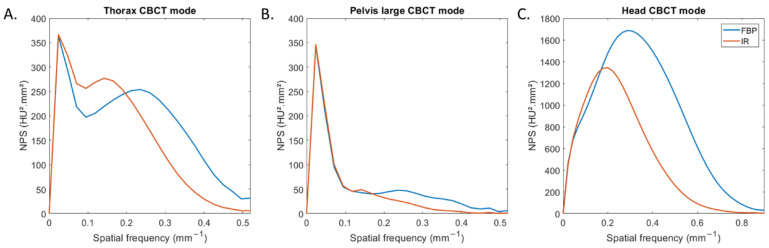
Noise power spectrum (NPS) curves obtained with filtered-back projection and iterative reconstruction for the three CBCT acquisition modes.

Figure 2 depicts the NPS curves obtained for all CBCT modes and both reconstruction types. For thorax and pelvis large modes, two NPS peaks were found: one placed at low spatial frequencies (0.02 mm^−1^) and the other at higher spatial frequencies (Figure 2A,B). For head mode, one NPS peak was found for both reconstruction types (Figure 2C).

For all CBCT acquisition modes, the spatial frequency of the NPS peak shifted towards lower frequency (f_peak_) with IR compared to FBP. f_peak_ decreased from 0.29 mm^−1^ to 0.20 mm^−1^ for head mode and from 0.22 mm^−1^ to 0.13 mm^−1^ for both other modes. For both reconstruction types, f_peak_ was higher for head mode than with the two other modes.

### 3.2. Task-Based Transfer Function

**Figure 3:**
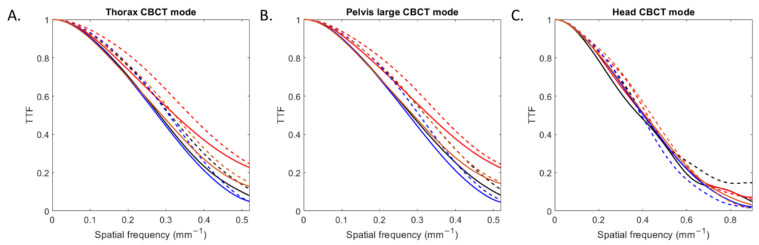
Task-based transfer function (TTF) curves of four inserts obtained with filtered-back projection and iterative reconstruction for the three CBCT acquisition modes.

The TTF curves obtained for all inserts according to the CBCT mode and reconstruction type are depicted in Figure 3. For all CBCT modes, TTF curves shifted towards higher spatial frequencies with IR compared to FBP. 

For all low and high-contrast inserts (Table 2), the values of the TTF at 50% (f_50_) increased with IR compared with FBP by an average of 5 ± 2% for head mode, 11 ± 1% for thorax mode and 11 ± 2% for pelvis large mode. For both reconstruction types, values of f_50_ were higher with head mode than with the other two modes. 

### 3.3. Contrast Value and Detectability Index

Contrast values and detectability indexes of the four inserts obtained for all CBCT modes and both reconstruction types are depicted in Table 2. 

For all modes and reconstruction types, the contrast values were similar for each insert. The mean contrast values were −1018 ± 10 HU for the air insert, −179 ± 2 HU for LDPE insert, 236 ± 2 HU for the Delrin insert and 793 ± 15 HU for the Teflon insert.

For all low and high-contrast simulated lesions, d’ values increased with IR than FBP for all CBCT modes. d’ values were higher by 55 ± 2% on average for head mode, 50 ± 6% for thorax mode and 81 ± 4% for pelvis large mode. For both reconstruction types, the highest d’ values were found for pelvis large mode and the lowest values for head mode.

### 3.4. Patients

**Figure 4:**
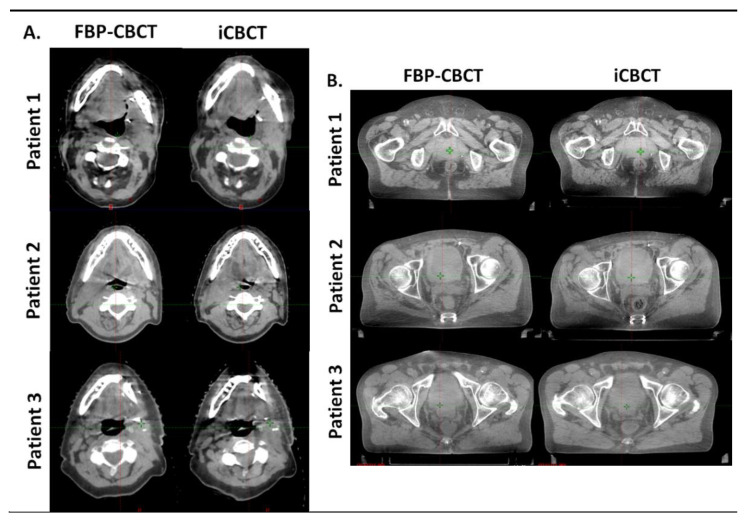
Comparison of head and neck (**A**) and pelvis (**B**) reconstruction with FBP (FBP-CBCT) and IR (iCBCT) on three patients. The series were matched on the same location and visualized with the same windowing.

Figure 4 shows the images obtained with FBP and IR for 3 patients on thorax CBCT acquisitions (Figure 4A) and in pelvis large CBCT mode (Figure 4B). For both anatomical locations, the images were less noisy and sharper (better visual border detection for all organs) with IR than with FBP. For the 6 patients, the technologists’ confidence in the CBCT vs reference planning CT (CT SIM) was equivalent for both types of CBCT image and the radiotherapist confirmed these results.

## 4. Discussion

For the first time, our study assessed the impact of the new iterative reconstruction (IR) algorithm available on the Halcyon^®^ V2.0 on CBCT images in comparison with CBCT images reconstructed with FBP. To assess the image quality of CBCT images, a task-based image quality assessment was performed. The use of IR reduced the image noise and improved spatial resolution and detectability, but changed the image texture by increasing image smoothness.

The outcomes of task-based image quality assessment confirmed the results found on conventional CT images with IR algorithms [5,6,7,19,20,21,22,24]. The NPS results showed that, for the 3 CBCT acquisition modes evaluated, the noise magnitude decreased with IR compared to FBP but the NPS curves shifted towards lower frequencies, resulting in more pronounced image smoothing. These results were also found on the 6 patients for the two anatomical locations where the images were less noisy but smoother with IR-CBCT. We also found that a second peak at low spatial frequency was positioned on the NPS curves for the thorax and wide pelvis CBCT acquisition modes. This peak was directly related to the presence of cupping effect artefacts on the FBP and IR images. This type of artefact is well-known [25] and corresponds to a defect in homogeneity of the CBCT images, related to several components [26,27] such beam hardening, beam softening and the high proportion of scattered photons on the large size detector. These artefacts were not visible on the 6 patients’ images. Furthermore, we found that the TTF curves of the four low- and high-contrast inserts shifted to higher spatial frequencies with IR than with FBP, resulting in improved spatial resolution. This improvement was confirmed visually with better image sharpness and visual border detection with IR for all organs than with FBP for all 6 patients. Last, we found that the detectability indexes of the four simulated lesions were better with IR than with FBP for all CBCT acquisition modes. These results are directly related to the noise magnitude and spatial resolution outcomes. As the contrast between the phantom’s background and the insert used to define each simulated lesion did not vary, the d’ values increased thanks to the strong decrease in noise and the improvement in spatial resolution with IR. Similar results were found for conventional CT images for different IR algorithms compared to FBP.

In clinical practice, the purpose of image-guided radiation therapy, is to improve target-matching on daily imagery with the reference planning CT (CT SIM). In this study, IR improved the image quality but only had a slight influence on patient care regarding matching CBCT to CT SIM. When discussing with technologists and physician, the problem was due to the beam-hardening artifact in the pelvic region, which was more pronounced with IR. In some cases, IR sequences also appeared to be over-smooth and the operator may have had difficulty delineating the borders of the structure. In this first version of iCBCT we do not have the possibility to set up the reconstruction. By the way, the user can simply tick or untick “iCBCT” on the imaging workstation. The possibility to play on the strength of smoothing could be an interesting improvement in the future. Moreover, the radiation protection safety principle of As Low As Reasonably Achievable (ALARA) should also be applied to the imaging dose in radiotherapy, minimizing imaging doses without compromising target matching. Indeed, the recent report by the AAPM Task Group 180 proposed recommendations to optimize imaging dosage [28]. The possibilities of dose reduction with the IR protocol whilst maintaining constant image quality seem interesting and should be investigated in future studies. Finally, in the future, IR could also prove to be essential during adaptive processing where a dosimetric calculation is carried out on the CBCT images of the day [24]. Indeed, it would be interesting to assess the impact of iCBCT on the elastic deformation in CT simulation as well as during the volume segmentation stage.

This study has certain limitations. Only one phantom was used for all acquisitions, therefore its size is not consistent with all protocols, and may deviate from the reality. Another point is that the use of detectability indices is questionable, as these are only partly adapted to the clinical task of radiotherapy. Indeed, the lesions are not always visible on CBCT. The aim is thus to detect the contours and not the lesions directly. In this sense, the TTF analysis was very important in our study, since it indirectly determines the quality of the contours. In other words, the higher the restitution of the contrast according to the spatial resolution, the more precise the contours, which facilitates subsequent registration.

## 5. Conclusions

This study shows that using the new iterative reconstruction algorithm available on the Halcyon^®^ V2.0 enhances the image quality of CBCT images by reducing image noise and improving the detectability and spatial resolution. These results offer interesting prospects for refining patient registration before treatment and boosting operator confidence. The improvement in detectability with IR also paves the way for reflection on the dose optimization of CBCT acquisitions. In any case, studies must be performed on a larger number of patients to confirm these results and their impact on patient registration accuracy.

## Figures and Tables

**Table 1:** Acquisition parameters used for each CBCT acquisition mode.

	kV	mA	Focal Spot (mm)	Exposure Time (s)	CTDI_vol_ (mGy)	FOV (mm)
Head	100	30	1	4.63	3.7	281.60
Thorax	125	35	1	8.59	6.0	492.37
Pelvis large	140	90	1	16.18	38.4	492.37

Footnotes: CTDI_vol_, volume CT dose index measured on the 32-cm diameter reference dosimetric phantom; FOV, field of view.

**Table 2:** Noise power spectrum, task-based transfer function, contrast values and detectability index outcomes obtained for all CBCT acquisition modes and reconstruction types.

	Head CBCT Mode		Thorax CBCT Mode		Pelvis Large CBCT Mode	
Metrics	FBP	IR	FBP	IR	FBP	IR
Square Root AUC NPS2D (HU)	40.0	26.0	10.6	8.6	4.8	3.6
f_peak_ (mm^−1^)	0.29	0.20	0.02/0.22	0.02/0.13	0.02/0.22	0.02/0.13
f_50_ Air (mm^−1^)	0.41	0.44	0.29	0.32	0.29	0.32
f_50_ LDPE (mm^−1^)	0.39	0.41	0.28	0.31	0.29	0.32
f_50_ Delrin (mm^−1^)	0.40	0.42	0.32	0.36	0.33	0.36
f_50_ Teflon (mm^−1^)	0.39	0.40	0.28	0.31	0.28	0.31
d’ Air	14.7	22.5	44.9	67.2	105.2	192.0
d’ LDPE	2.5	4.0	8.0	11.6	18.5	33.4
d’ Delrin	3.4	5.3	10.5	16.7	25.1	46.7
d’ Teflon	11.8	18.1	35.2	51.5	81.6	143.4
Contrast Air (HU)	−1005.6	−1004.5	−1024.0	−1023.0	−1025.0	−1024.8
Contrast LDPE (HU)	−181.3	−180.8	−178.3	−178.6	−177.9	−178.0
Contrast Delrin (HU)	237.7	239.4	234.7	236.5	233.1	234.4
Contrast Teflon (HU)	808.3	812.8	786.8	792.0	776.1	781.0

Footnote: FBP: filtered back projection; IR: iterative reconstruction.

## Data Availability

The data presented in this study are available on request from the corresponding author reasonable request.

## References

[1] Wiersma R.D., Mao W., Xing L. (2008). Combined kV and MV imaging for real-time tracking of implanted fiducial markers. Med. Phys..

[2] Leech M., Coffey M., Mast M., Moura F., Osztavics A., Pasini D., Vaandering A. (2017). ESTRO ACROP guidelines for positioning, immobilisation and position verification of head and neck patients for radiation therapists. Tech. Innov. Patient Support Radiat. Oncol..

[3] Nagarajappa A.K., Dwivedi N., Tiwari R. (2015). Artifacts: The downturn of CBCT image. J. Int. Soc. Prev. Community Dent..

[4] Verdun F.R., Racine D., Ott J.G., Tapiovaara M.J., Toroi P., Bochud F.O., Veldkamp W.J.H., Schegerer A., Bouwman R.W., Giron I.H. (2015). Image quality in CT: From physical measurements to model observers. Phys. Med..

[5] Samei E., Richard S. (2015). Assessment of the dose reduction potential of a model-based iterative reconstruction algorithm using a task-based performance metrology. Med. Phys..

[6] Greffier J., Frandon J., Larbi A., Beregi J.P., Pereira F. (2020). CT iterative reconstruction algorithms: A task-based image quality assessment. Eur. Radiol..

[7] Greffier J., Frandon J., Pereira F., Hamard A., Beregi J.P., Larbi A., Omoumi P. (2020). Optimization of radiation dose for CT detection of lytic and sclerotic bone lesions: A phantom study. Eur. Radiol..

[8] Richard S., Husarik D.B., Yadava G., Murphy S.N., Samei E. (2012). Towards task-based assessment of CT performance: System and object MTF across different reconstruction algorithms. Med. Phys..

[9] Samei E., Bakalyar D., Boedeker K.L., Brady S., Fan J., Leng S., Myers K.J., Popescu L.M., Ramirez Giraldo J.C., Ranallo F. (2019). Performance evaluation of computed tomography systems: Summary of AAPM Task Group 233. Med. Phys..

[10] Gang G.J., Lee J., Stayman J.W., Tward D.J., Zbijewski W., Prince J.L., Siewerdsen J.H. (2011). Analysis of Fourier-domain task-based detectability index in tomosynthesis and cone-beam CT in relation to human observer performance. Med. Phys..

[11] Zbijewski W., De Jean P., Prakash P., Ding Y., Stayman J.W., Packard N., Senn R., Yang D., Yorkston J., Machado A. (2011). A dedicated cone-beam CT system for musculoskeletal extremities imaging: Design, optimization, and initial performance characterization. Med. Phys..

[12] Christianson O., Chen J.J., Yang Z., Saiprasad G., Dima A., Filliben J.J., Peskin A., Trimble C., Siegel E.L., Samei E. (2015). An Improved Index of Image Quality for Task-based Performance of CT Iterative Reconstruction across Three Commercial Implementations. Radiology.

[13] Katsura M., Matsuda I., Akahane M., Sato J., Akai H., Yasaka K., Kunimatsu A., Ohtomo K. (2012). Model-based iterative reconstruction technique for radiation dose reduction in chest CT: Comparison with the adaptive statistical iterative reconstruction technique. Eur. Radiol..

[14] Larbi A., Orliac C., Frandon J., Pereira F., Ruyer A., Goupil J., Macri F., Beregi J.P., Greffier J. (2018). Detection and characterization of focal liver lesions with ultra-low dose computed tomography in neoplastic patients. Diagn. Interv. Imaging.

[15] Macri F., Greffier J., Pereira F., Rosa A.C., Khasanova E., Claret P.G., Larbi A., Gualdi G., Beregi J.P. (2016). Value of ultra-low-dose chest CT with iterative reconstruction for selected emergency room patients with acute dyspnea. Eur. J. Radiol..

[16] Yamada Y., Jinzaki M., Hosokawa T., Tanami Y., Sugiura H., Abe T., Kuribayashi S. (2012). Dose reduction in chest CT: Comparison of the adaptive iterative dose reduction 3D, adaptive iterative dose reduction, and filtered back projection reconstruction techniques. Eur. J. Radiol..

[17] Yan C., Xu J., Liang C., Wei Q., Wu Y., Xiong W., Zheng H., Xu Y. (2018). Radiation Dose Reduction by Using CT with Iterative Model Reconstruction in Patients with Pulmonary Invasive Fungal Infection. Radiology.

[18] Greffier J., Barbotteau Y., Gardavaud F. (2022). iQMetrix-CT: New software for task-based image quality assessment of phantom CT images. Diagn. Interv. Imaging.

[19] Greffier J., Si-Mohamed S., Frandon J., Loisy M., de Oliveira F., Beregi J.P., Dabli D. (2022). Impact of an artificial intelligence deep-learning reconstruction algorithm for CT on image quality and potential dose reduction: A phantom study. Med. Phys..

[20] Greffier J., Frandon J., Durand Q., Kammoun T., Loisy M., Beregi J.P., Dabli D. (2022). Contribution of an artificial intelligence deep-learning reconstruction algorithm for dose optimization in lumbar spine CT examination: A phantom study. Diagn. Interv. Imaging.

[21] Greffier J., Durand Q., Frandon J., Si-Mohamed S., Loisy M., de Oliveira F., Beregi J.P., Dabli D. (2022). Improved image quality and dose reduction in abdominal CT with deep-learning reconstruction algorithm: A phantom study. Eur. Radiol..

[22] Greffier J., Dabli D., Frandon J., Hamard A., Belaouni A., Akessoul P., Fuamba Y., Le Roy J., Guiu B., Beregi J.P. (2021). Comparison of two versions of a deep learning image reconstruction algorithm on CT image quality and dose reduction: A phantom study. Med. Phys..

[23] Eckstein M., Bartroff J., Abbey C., Whiting J., Bochud F. (2003). Automated computer evaluation and optimization of image compression of x-ray coronary angiograms for signal known exactly detection tasks. Opt. Express.

[24] Racine D., Becce F., Viry A., Monnin P., Thomsen B., Verdun F.R., Rotzinger D.C. (2020). Task-based characterization of a deep learning image reconstruction and comparison with filtered back-projection and a partial model-based iterative reconstruction in abdominal CT: A phantom study. Phys. Med..

[25] Octave N. (2015). La Radiothérapie Adaptative et Guidée par Imagerie Avec la Technologie Cone-Beam CT: Mise en Oeuvre en vue du Traitement de la Prostate. Doctoral Dissertation.

[26] Hunter A.K., McDavid W.D. (2012). Characterization and correction of cupping effect artefacts in cone beam CT. Dentomaxillofac. Radiol..

[27] Huger S. (2013). Adaptation Interactive d’un Traitement de Radiothérapie par Imagerie Volumique: Développement et Validation d’outils pour sa Mise en Œuvre en Routine Clinique. Doctoral Dissertation.

[28] Glide-Hurst C.K., Lee P., Yock A.D., Olsen J.R., Cao M., Siddiqui F., Parker W., Doemer A., Rong Y., Kishan A.U. (2021). Adaptive Radiation Therapy (ART) Strategies and Technical Considerations: A State of the ART Review From NRG Oncology. Int. J. Radiat. Oncol. Biol. Phys..

